# A giant quiet esophageal polyp: A case report

**DOI:** 10.1002/ccr3.6823

**Published:** 2023-01-11

**Authors:** Hossein Sadidi, Ghazale Ahmadi, Ali Mehri, Marzie Nouri Dalouee

**Affiliations:** ^1^ Department of Thoracic Surgery Faculty of Medicine, Mashhad University of Medical Sciences Mashhad Iran; ^2^ Endoscopic and Minimally Invasive Surgery Research Center Mashhad University of Medical Sciences Mashhad Iran; ^3^ Cardiothoracic Surgery and Transplant Research Center Emam Reza Hospital, Faculty of Medicine, Mashhad University of Medical Sciences Mashhad Iran

**Keywords:** esophagus, fibrovascular, polyp

## Abstract

Fibrovascular polyps of the esophagus are rare, benign, intraluminal submucosal tumors. In this report, we present a case of esophageal polyp in a young woman and discuss its diagnostic and surgical aspects.

## Abstract 2

Esophageal polyps are rare and asymptomatic; however, they can grow extensively and cause significant discomfort for the patient. Therefore, it is crucial to consider them as a differential diagnosis.

## 1 INTRODUCTION

Fibrovascular polyps of the esophagus are rare, benign, intraluminal submucosal tumors that usually originate from the proximal esophagus and are regarded as 0.5%–1% of benign esophageal lesions.^1^, ^2^, ^3^ These lesions are typically individual and mainly affect men aged 53 years on average, with a range of 1–88 years.^4^, ^5^ Fibrovascular polyps are generally asymptomatic and small, usually detected incidentally during endoscopy.^5^ These tumors may expand to enormous sizes after exhibiting slow growth for a long time, causing prominent digestive or respiratory discomfort.^6^ The most common symptoms are dysphagia and a sensation of a mass in the neck. Other signs and symptoms include retrosternal or epigastric discomfort, weight loss, odynophagia, vomiting, and respiratory symptoms such as persistent cough and dyspnea.^4^ Fibrovascular polyps are associated with several complications, notably regurgitation of the poly, which is potentially life‐threatening and may cause asphyxia and laryngeal obstruction, leading to sudden death.^7^, ^8^ In addition, hemorrhage and necrosis of the lesion could occur if the polyp twists. Therefore, surgical removal of a fibrovascular polyp is indicated.^9^ We hereby report a case of a giant esophageal fibrovascular polyp in a young female patient that was resected surgically.

## 2 CASE PRESENTATION

**FIGURE 1:**
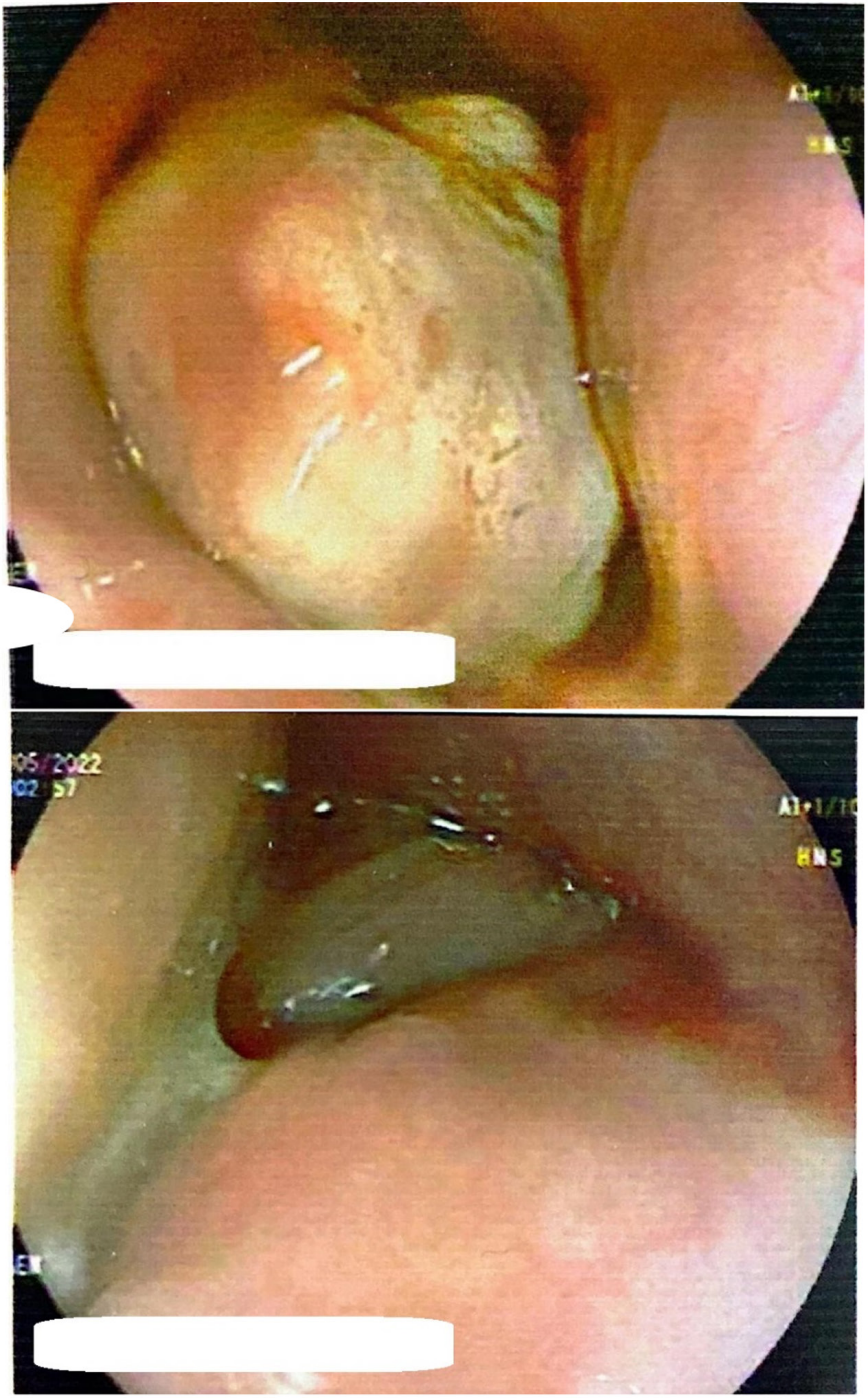
Endoscopic images of the esophageal polyp

**FIGURE 2:**
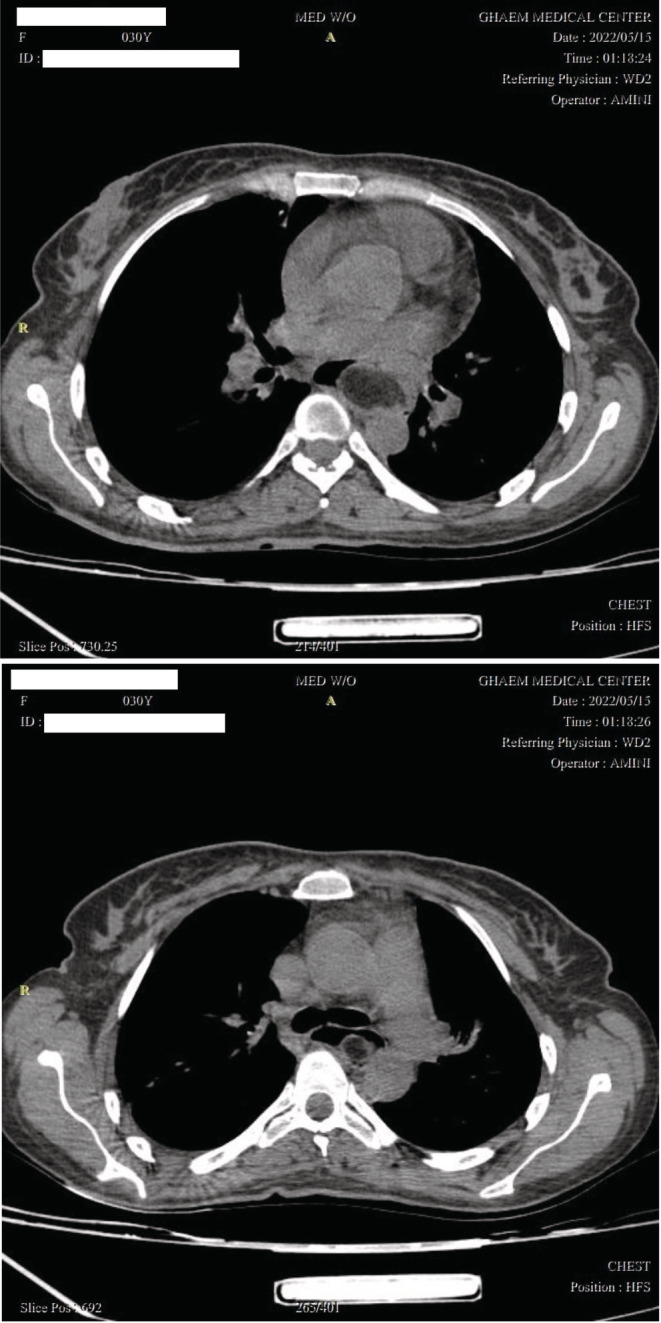
Axial views of the esophageal polyp CT scan

A 30‐year‐old woman with a history of cholecystectomy and ovarian cystectomy presented due to dysphagia to solid food and a foreign body sensation in her throat since childhood. Her symptoms had gradually progressed to the point that she also experienced difficulty swallowing liquids. Initially, a chest X‐ray was obtained, which was found to be normal. In the next step, she underwent upper GI endoscopy, where a giant intraluminal mass in her esophagus was detected (Figure 1). Spiral lung CT scan results showed a pedunculated submucosal intraluminal mass with fatty density, originating from the upper thoracic esophagus and extending down to the middle thoracic esophagus, suggestive of a fibrovascular polyp (Figure 2). Subsequently, the patient was referred to our surgery clinic for resection of the intraesophageal mass.

**FIGURE 3:**
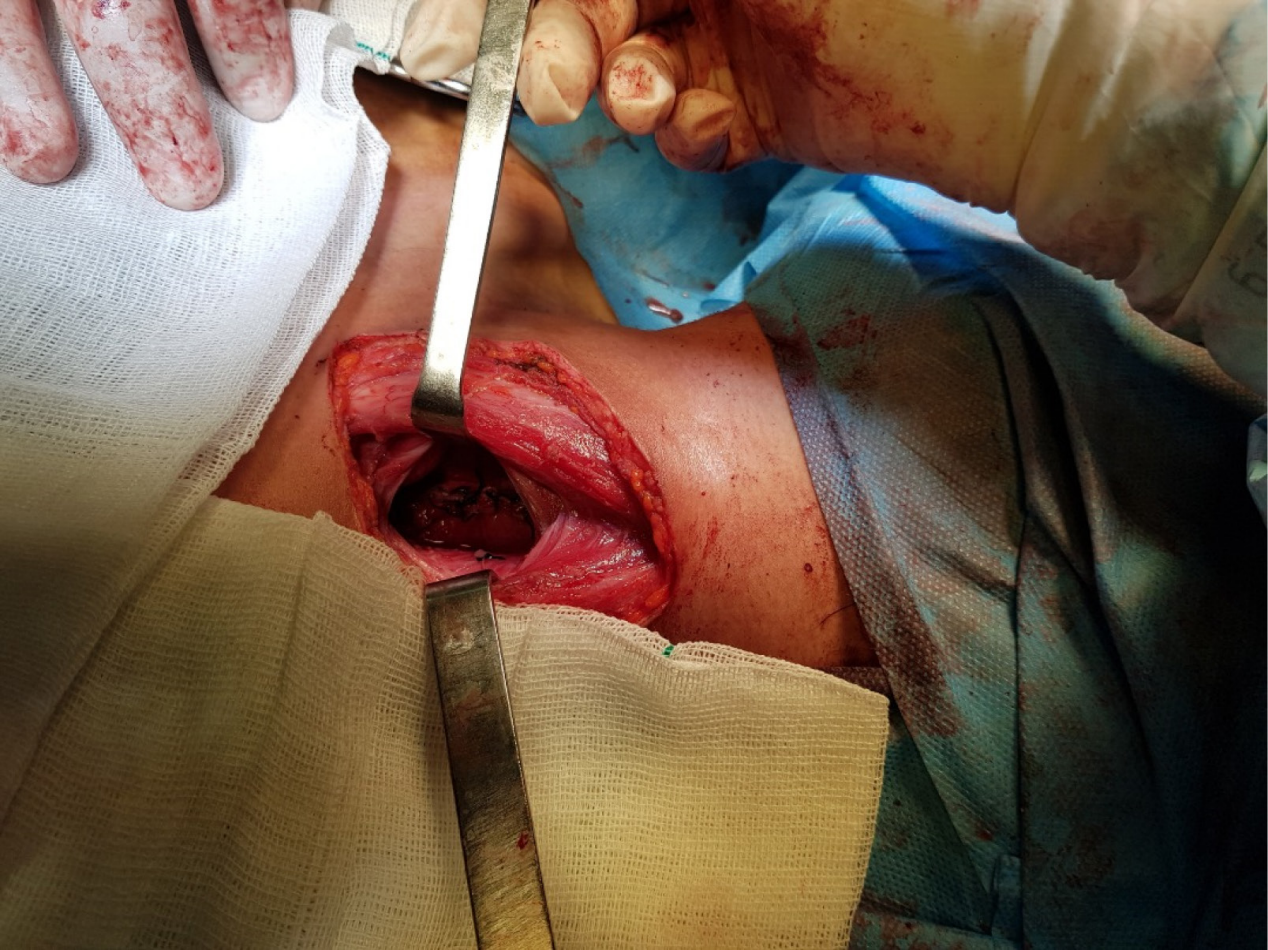
Surgical exploration of the polyp

**FIGURE 4:**
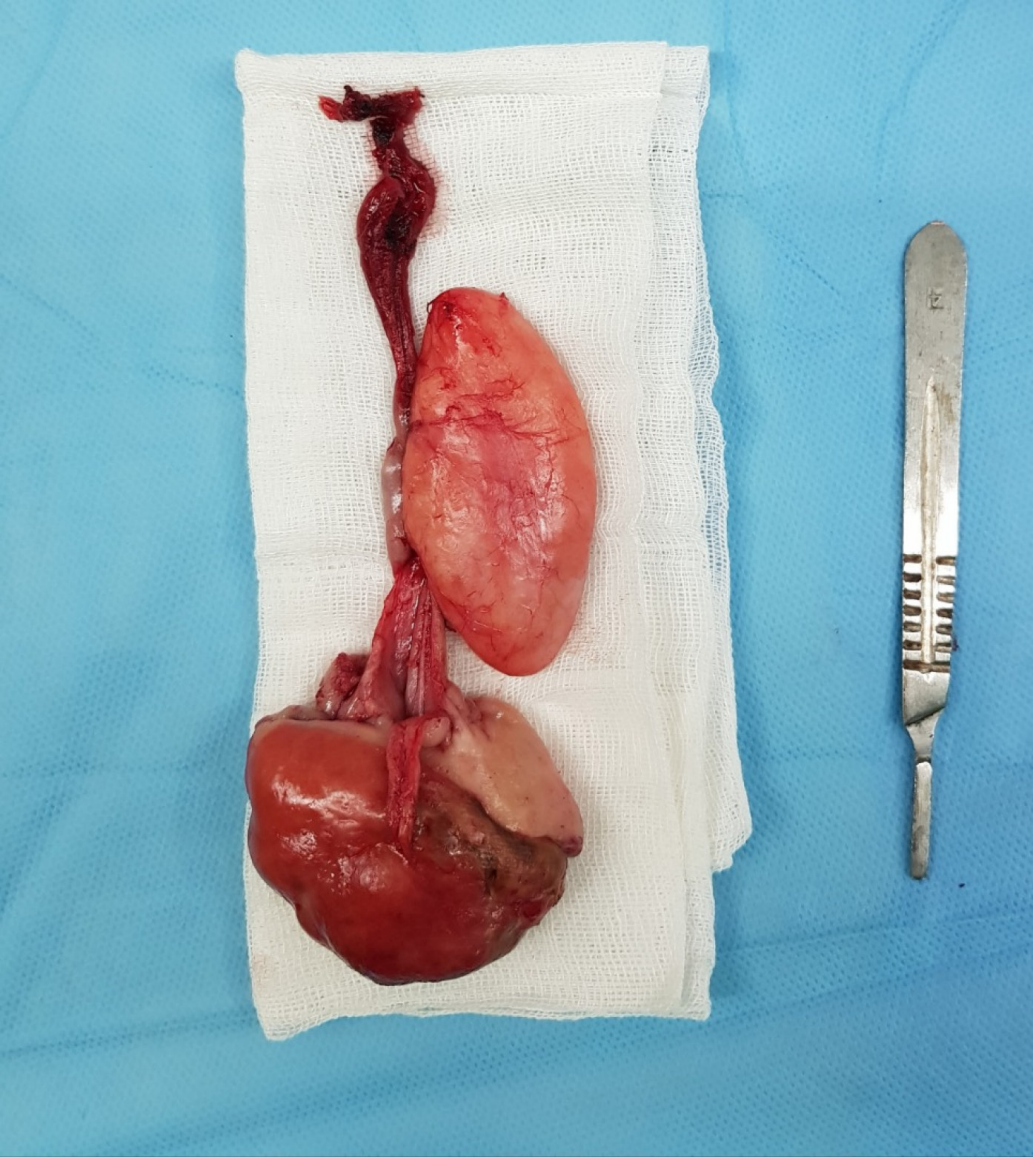
Resected esophageal polyp

After failing to determine the location of the polyp using a rigid esophagoscope, surgical exploration was performed by a left‐sided cervical esophagotomy (Figure 3). After the dissection of muscles, the esophagus dehisced from the sides with caution. After a longitudinal cut on the anterior of the esophagus, internal esophageal dissection was performed. The polyp base was fully exposed, and the 5 × 15‐centimeter white color mass was successfully resected. Finally, the esophagus was repaired in two layers longitudinally. Pathologic evaluation of resected sections revealed a cream‐yellow pedunculated polypoid structure with elastic consistency measuring 0.4 x 6 cm (Figure 4), lined by fibrinolytic exudate and granulation tissue formation with edematous stroma and numerous blood vessels with some inflammatory cells.

**FIGURE 5:**
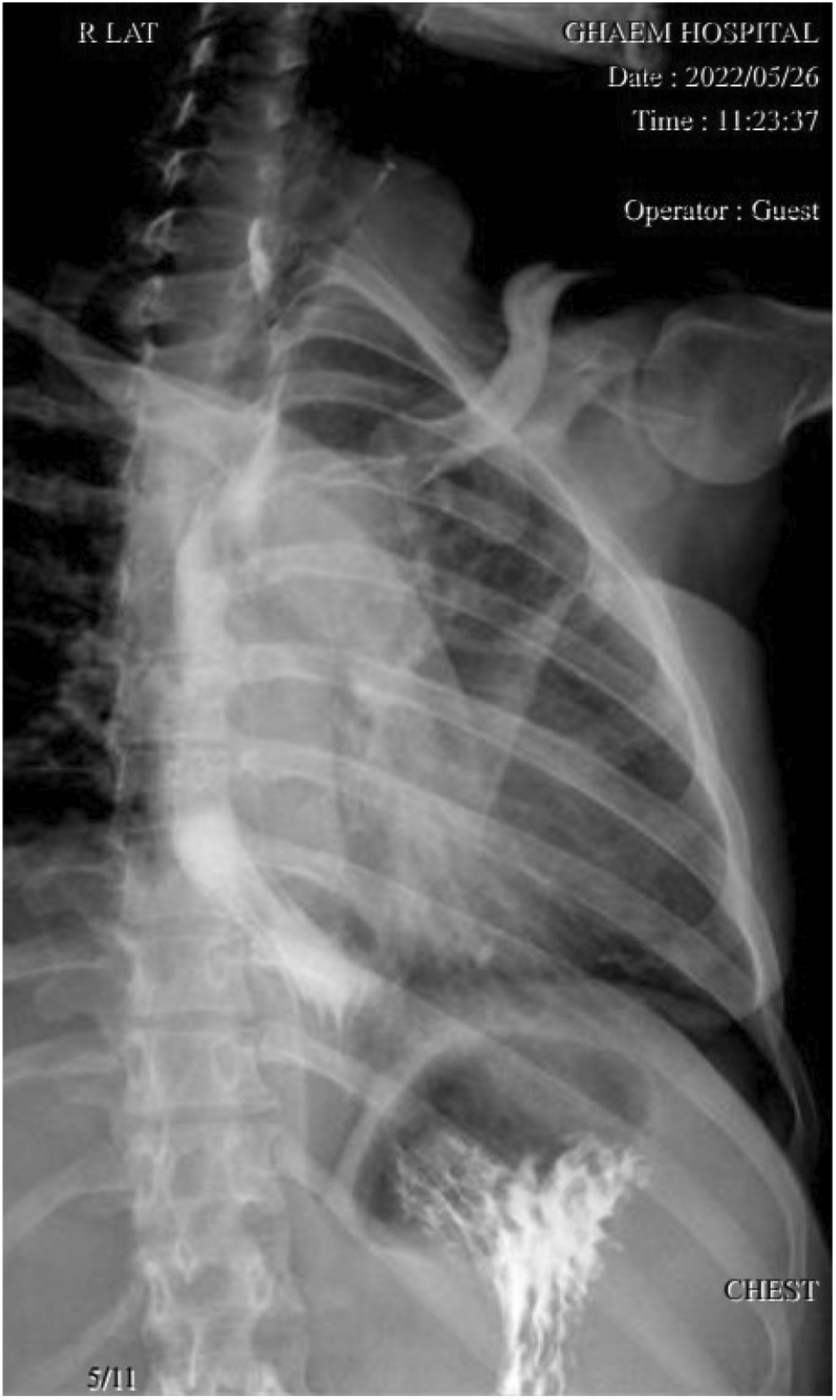
Post‐up barium swallow study

She spent 1 day in ICU and 4 days in the inpatient ward with a drain. The patient's post‐operation recovery was uneventful. A chest radiograph was obtained after surgery, which was normal. The post‐up barium swallow study showed only a mild dilation in the thoracic esophagus, with no evidence of leakage or extravasation (Figure 5). Eventually, she was discharged to her home in good condition.

## 3 DISCUSSION

Esophageal fibrovascular polyps are benign tumors of the upper digestive tract. Few reports of malignant transformation of these tumors exist.^10^ However, sarcomas and squamous carcinomas can originate from the esophageal polyp's lipomatous component and mucosa, respectively.^11^

Fibrovascular polyps usually arise as small mucosal tumors but gradually increase in size due to constant downward pressure by peristalsis and food matter.^2^ The slow‐growing nature of fibrovascular polyps delays the onset of symptoms and may prolong the time to diagnosis. Large polyps may be associated with life‐threatening complications such as asphyxia and laryngeal obstruction by the polyp.^7^, ^8^ False negative reports of endoscopic studies could occur in cases where the mass size is smaller than 2 cm in diameter, leading to delayed diagnosis.^12^

Fibrovascular polyps usually require surgery, but the type and route of surgical intervention depend on the pedicle's origin, size, and vascularity.^2^ Besides surgical mass removal, endoscopic resection could be utilized for polyps smaller than 2 cm in diameter with a thin pedicle. Also, there is a report of endoscopic removal of a giant esophageal fibrovascular polyp.^13^ Based on the visibility of the polyp stalk, peroral excision with the assistance of electrocautery is also an option. Owens et al. successfully performed the lateral pharyngotomy technique for large polyps in their report of two patients.^10^ Also, Hoseok et al. used a bi‐approach surgical treatment (transcervical and transabdominal approach simultaneously) for the excision of a giant fibrovascular polyp of the esophagus.^14^

## 4 CONCLUSIONS

Although esophageal polyps are rare and primarily asymptomatic, they may grow extensively and cause significant discomfort for the patient, necessitating surgical intervention. Consequently, they should be considered as a differential diagnosis.

## AUTHOR CONTRIBUTIONS

**Hossein Sadidi:** Resources; supervision; writing – review and editing.

## CONFLICT OF INTEREST

The authors have no conflicts of interest to declare.

## ETHICAL APPROVAL

Written informed consent was obtained from the patient's parent to publish this report in accordance with the journal's patient consent policy.

## CONSENT

Written informed consent was obtained from the patient to publish this report in accordance with the journal's patient consent policy.

## Data Availability

Data sharing not applicable to this article as no datasets were generated or analysed during the current study.
